# Blue emitting exciplex for yellow and white organic light-emitting diodes

**DOI:** 10.1007/s12200-023-00101-3

**Published:** 2023-12-14

**Authors:** Kavya Rajeev, C. K. Vipin, Anjali K. Sajeev, Atul Shukla, Sarah K. M. McGregor, Shih-Chun Lo, Ebinazar B. Namdas, K. N. Narayanan Unni

**Affiliations:** 1 Centre for Sustainable Energy Technologies, CSIR-National Institute for Interdisciplinary Science and Technology, Thiruvananthapuram, 695 019 India; 2 Academy of Scientific and Innovative Research (AcSIR), Ghaziabad, 201002 India; 3 Centre for Organic Photonics & Electronics, The University of Queensland, Brisbane, QLD 4072 Australia; 4 School of Mathematics and Physics, The University of Queensland, Brisbane, QLD 4072 Australia; 5 School of Chemistry and Molecular Biosciences, The University of Queensland, Brisbane, QLD 4072 Australia

**Keywords:** Organic light-emitting diodes, Dual functional exciplex, Spacers, Device design strategy, Blue exciplex, Yellow OLEDs, White light emission

## Abstract

White organic light-emitting diodes (WOLEDs) have several desirable features, but their commercialization is hindered by the poor stability of blue light emitters and high production costs due to complicated device structures. Herein, we investigate a standard blue emitting hole transporting material (HTM) *N*,*N'*-bis(naphthalen-1-yl)-*N*,*N'*-bis(phenyl)benzidine (NPB) and its exciplex emission upon combining with a suitable electron transporting material (ETM), 3-(biphenyl-4-yl)-5-(4-*tert*-butylphenyl)-4-phenyl-4*H*-1,2,4-triazole (TAZ). Blue and yellow OLEDs with simple device structures are developed by using a blend layer, NPB:TAZ, as a blue emitter as well as a host for yellow phosphorescent dopant iridium (III) bis(4-phenylthieno[3,2-*c*]pyridinato-N,C^2^^'^)acetylacetonate (PO-01). Strategic device design then exploits the ambipolar charge transport properties of tetracene as a spacer layer to connect these blue and yellow emitting units. The tetracene-linked device demonstrates more promising results compared to those using a conventional charge generation layer (CGL). Judicious choice of the spacer prevents exciton diffusion from the blue emitter unit, yet facilitates charge carrier transport to the yellow emitter unit to enable additional exciplex formation. This complementary behavior of the spacer improves the blue emission properties concomitantly yielding reasonable yellow emission. The overall white light emission properties are enhanced, achieving CIE coordinates (0.36, 0.39) and color temperature (4643 K) similar to daylight. Employing intermolecular exciplex emission in OLEDs simplifies the device architecture via its dual functionality as a host and as an emitter.

**Graphical abstract:**

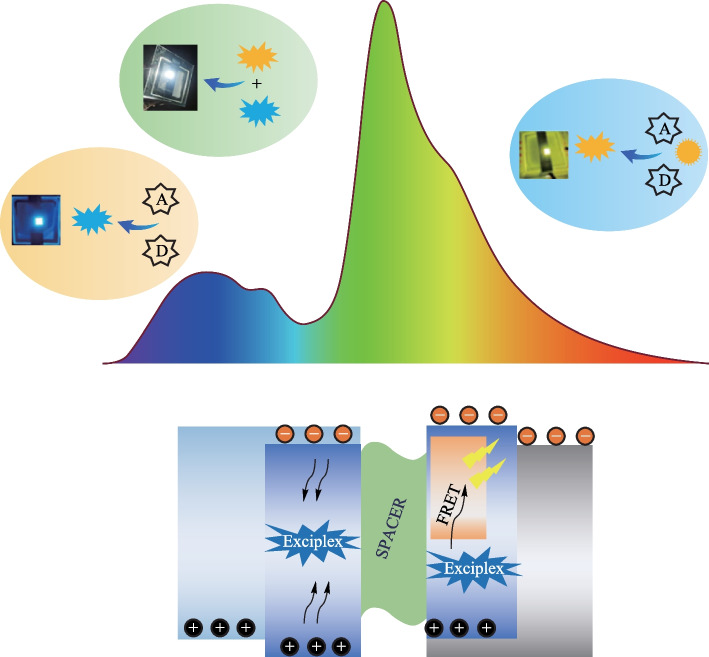

**Supplementary Information:**

The online version contains supplementary material available at 10.1007/s12200-023-00101-3.

## Introduction

OLEDs are one of the major components in the smart electronic world. Smart displays, watches, and other electronic display gadgets employing OLEDs have an ubiquitous presence in everyday life. After the first OLED was reported in 1987 [1], rapid development enabled commercial production of OLED displays by 1997, with the technology making steady progress ever since. However, the high cost and limited life span of OLED products are considered to be the major challenges preventing their deeper market penetration. These are being addressed by efficient out-coupling techniques [2–4], novel emitter molecules [5, 6], efficient emission mechanisms such as phosphorescence [7], thermally activated delayed fluorescence (TADF) [8] and simplified device architectures [9]. However, stable and efficient blue emission is still considered a bottleneck in the OLED display industry. This is due to the challenges associated with molecular design of wide band gap materials. Unfortunately, these design challenges are present across fluorescent, phosphorescent, and TADF materials, resulting in a scarcity of suitable materials. Furthermore, the lack of stable blue fluorophores is detrimental to application in RGB color balancing of displays. Hence, there is an urgent need to further develop stable and efficient blue emitters and to improve the yield of existing materials.

There are many blue emitting charge transport materials, which have not been explored due to their low quantum yield. Hence, novel device designs utilizing commonly available blue emitting transport molecules would provide a major breakthrough. In the past few years, excited state emission mechanisms such as exciplex (involving excited states in pairings of materials) have emerged as an alternative resource for use in OLEDs. Exciplex emission occurs at the interface of an HTM and an ETM and it provides a simple device architecture by avoiding the need for a separate emissive layer (EML). Exciplex as an emission mechanism has comparatively low quantum yield. Nonetheless, it can be effectively used as a host with phosphorescent, fluorescent [10] and TADF dopants [11]. Suitable exciplex combinations with novel design strategies can provide new routes for efficient white OLEDs (WOLEDs) without the need for complicated tandem structures. However, establishing a general criterion for the selection of conjugate pairs for efficient exciplex emission remains a complex issue. Exciplex emission can occur via electrical as well as optical excitation. The basic criterion for the selection of materials is to have a moderate offset [12] between the highest occupied molecular orbital (HOMO) energies of the HTM and ETM. Lowest unoccupied molecular orbital (LUMO) energies are also expected to have a similar offset. Exciplex emission, previously thought of as a less efficient process, has, since 2000, regained a role in OLEDs as an emitter as well as a host [13]. Most of the reported high efficiency exciplex OLEDs have utilized the exciplex as a host rather than as an emitter. Although the quantum yield for exciplex emission is quite low, proper selection of conjugate pairs can provide adequate intensity of emission.

In this work, we have addressed the issue of lack of blue emitters in OLEDs by utilizing an intermolecular excited state formed at the interface of charge transporting materials; NPB and TAZ. This exciplex was used as a blue emitter as well as a host material for a yellow emitting phosphorescent dopant (PO-01) OLEDs. An external quantum efficiency (EQE) of (6.9 ± 0.27) % @ 1000 cd/m^2^ was obtained for the yellow OLED. Furthermore, white emission with CIE coordinates (0.36, 0.39) was achieved by employing tetracene as a spacer in conjunction with blue exciplex host and yellow emitting phosphorescent dopant.

## Methods

**Fig. 1:**
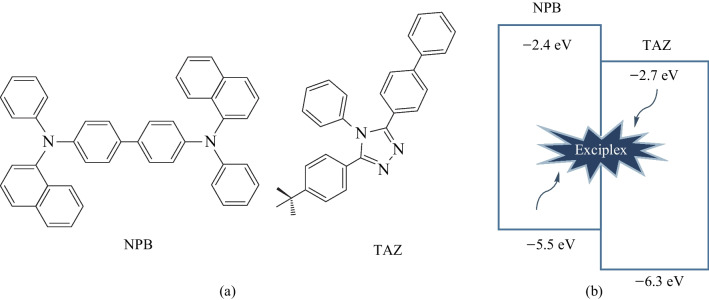
**a** Molecular structures of NPB and TAZ. **b** Comparison of energy level (in eV) diagrams and mechanism of exciplex formation

The molecular structures of NPB, and TAZ are shown in Fig. 1a. Solid-state photophysical measurements were performed on thin films deposited on fused silica substrates. Substrates were cleaned with acetone and isopropanol followed by UV-ozone to remove organic impurities. Photoluminescence (PL) spectra were measured using a FS5 fluorescence spectrometer (Edinburgh Instruments). Absolute photoluminescence quantum yields (PLQYs) for thin film samples were measured using the same spectrometer with calibrated integrating sphere. PLQY/PL spectra of NPB and NPB:TAZ blend films were measured by exciting at 350 nm, while neat films of TAZ were excited at 300 nm. Emission lifetime was measured with the same spectrometer, and samples were excited at 375 nm using a laser diode with an instrument response function (IRF) of 150 ps.

Devices were fabricated in a nitrogen glove box-integrated thermal evaporation system (Angstrom Inc.) and the film thickness was measured using Dektak XT profilometer. Indium tin oxide (ITO) substrates were purchased from Kintec Company, Hong Kong, China and organic materials were purchased from Luminescence Technology Corp. (Lumtec), Taiwan, China. The substrates were cleaned by using a liquid detergent followed by sequential sonication in isopropanol and de-ionized water for 15 min each. After drying, the UV-ozone treated (Novascan) substrates were loaded in thermal evaporation chamber. All the layers (materials) of the device were deposited on the substrate by thermal evaporation under high vacuum (≈ 10^−7^ torr) conditions. After the evaporation, the devices were encapsulated inside the nitrogen filled glovebox by using a UV-curable epoxy (Epoxy Technology Inc.). The OLED characterization system consists of a SpectraScan PR-655 spectroradiometer integrated with a Keithely 2400 sourcemeter.

## Results and discussion

### Device fabrication and characterization

#### Blue OLEDs by combining NPB and TAZ

**Fig. 2:**
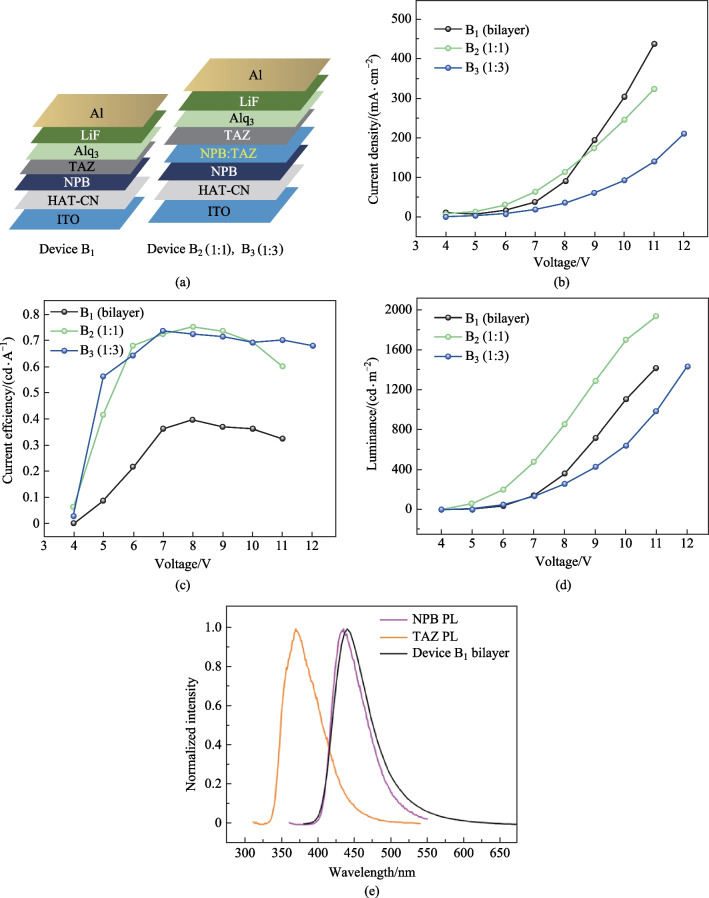
**a** OLED architectures of NPB/TAZ bilayer (B_1_) and NPB:TAZ (1:1 and 1:3) blend devices (for B_2_ and B_3_, respectively). **b** Current density vs voltage plot. **c** Current efficiency vs voltage plot. **d** Luminance vs voltage plot; **e** Comparison of photoluminescence and electroluminescence of NPB/TAZ bilayer OLED B_1_ devices

Here we combined NPB with TAZ to create a blue emitting unit. Devices fabricated by combining NPB with TAZ could yield: excitonic emission of NPB; energy transfer between the ETL and NPB; or exciplex emission at the NPB/ETL interface. We performed spectroscopic studies of the thin films to confirm the dominant emission mechanism in photoluminescence (PL). However, it was also critical to determine the dominant mechanism in electroluminescence and further improve the overall efficiency. The HOMO–LUMO offset values at the NPB/TAZ interface were also compared (see Fig. 1b) [14, 15]. The exciplex emission is usually favored for HOMO-HOMO/LUMO-LUMO gap ≥ 0.4 eV [16]. For the NPB:TAZ combination, the offset values are below 0.4 eV. Hence, a bilayer NPB/TAZ device (B_1_) was fabricated with a device architecture of ITO/HAT-CN (5 nm)/NPB (60 nm)/TAZ (40 nm)/Alq_3_ (20 nm)/LiF (1 nm)/Al (100 nm) as shown in Fig. 2a, where HAT-CN is 1,4,5,8,9,11-hexaazatriphenylenehexacarbonitrile. A thin layer of HAT-CN (5 nm) was deposited prior to NPB to improve the hole injection into NPB. To balance the hole injection into TAZ, Alq_3_ was incorporated in the structure, as an electron transport layer (ETL). To get better insights into energy transfer in the process of electroluminescence, we further fabricated devices (B_2_ and B_3_) with NPB:TAZ as the EML sandwiched by the pristine NPB and TAZ layers. The NPB and TAZ layers on both sides provided better charge transport and carrier confinement. Specifically, EML consists of the co-deposited layer of NPB:TAZ at 1:1 and 1:3 for devices B_2_ and B_3_, respectively, with total thickness of 15 nm. However, the doping ratio of NPB in TAZ in device B_3_ was reduced by three times compared to B_2_.

The electroluminescence of the OLEDs with NPB/TAZ bilayer and blend layer (NPB:TAZ) were analyzed via device characteristics as shown in Fig. 2b, c, d. Interestingly, the current density of the bilayer device increased compared to blend devices at higher voltages as can be seen from Fig. 2b. In the bilayer device, the energy barrier for hole injection from NPB to TAZ was 0.8 eV. At higher voltages this barrier was overcome and the increased hole injection and subsequent recombination (radiative or nonradiative) may have caused the current. It may be noted that the blend device was nothing but a device where a 15 nm blend layer had been added in between the pristine NPB and TAZ layers of the bilayer device. At higher voltages, injection to the blend was increased but charges may have become trapped in the blend layer. In bilayer, this does not happen as the charges recombine either radiatively or non-radiatively at the interface. However, the trapping of carriers in the blend may have helped the exciplex formation. To study this, we looked at the luminanace (*L*) vs current density (*J*) plots of these devices as shown in Fig. S1 in the Supplementary Information (SI) and found out that compared to the blend devices, bilayer device had a much higher *J* value for the same *L* value. We believe that the radiative recombination even in the thin layer of blend layer was much more efficient than the bilayer device. However overall *J*, which is the sum of radiative and nonradiative currents could have been higher for the bilayer device, particularly at higher voltages.

The current density–voltage and current efficiency–voltage plots of the devices were compared. From the device characteristics, the luminance was seen to be enhanced when NPB:TAZ layer was incorporated. As shown in Fig. 2c, the current efficiency of B_2_ and B_3_ devices increased to 0.7 cd/A compared to that of bilayer NPB/TAZ B_1_ devices, an enhancement by a factor of 2 for the NPB:TAZ blend devices. Given that exciton recombination could occur within NPB or at the NPB/TAZ interface, the effect of TAZ at the interface of NPB was investigated in detail through photophysical studies of the thin films of individual materials and their co-evaporated films, which was essential to understand the origin of PL. Hence, spectroscopic studies were carried out to investigate whether the emission was due to energy transfer or exciplex emission in the mixed layer of NPB/TAZ. The steady-state absorption and emission spectra of the molecules were studied in the thin film state. The UV–Visible absorption spectra, as well as the emission spectra of the films of TAZ, NPB and NPB:TAZ (1:1, 1:3), are shown in Fig. S2a and b in the SI. The comparison of PL of neat films with the EL spectra of the bilayer device is shown in Fig. 2e. The emission from TAZ can be completely ruled out as its emission spectrum was significantly different from the EL spectra. The emission spectra of NPB and blend films were not identical. The full width at half maximum (FWHM) of the PL peaks of the films of NPB, TAZ, and NPB:TAZ were 52, 54, and 63 nm, respectively. The fluorescence decay parameters of NPB, TAZ and NPB:TAZ films are shown in Table S1 in the SI. A slight red shifted emission with increase in FWHM was observed for the blend films but was not observed for the neat films. Exciplex usually leads to the red shifted emission and broadened spectrum compared to those of the individual acceptor or donor molecule. Hence, this broadness of emission in the blend films can be attributed to the exciplex formation. However, considering the absorption spectrum of NPB and photoluminescence spectrum of TAZ, energy transfer also seems likely, as there is sufficient overlap between the emission and absorption spectra of neat films of TAZ and NPB, respectively. As is shown, Fig. S2b in the SI indicates a possibility of Förster resonance energy transfer (FRET) from TAZ to NPB. We compared the PL of the NPB:TAZ blend films at two different ratios 1:1 and 1:3. The PL of TAZ was completely quenched in both 1:1 and 1:3 blend films. The PLQYs of the 1:1 and 1:3 blend films were found to be 38% and 44%, respectively. A slight enhancement in PLQYs for the 1:3 blend films again showed the possibility of energy transfer from TAZ to NPB. The transient emission properties of the blend films were found similar to that of NPB’s PL as shown in Fig. S3a in the SI, with the values compared in Table S2 in the SI. Therefore, the results suggest that the energy transfer from TAZ to NPB resulted in the blue excitonic emission from NPB under optical excitation. To confirm this, we needed to rule out the chances for exciplex formation; we further studied the transient PL of the blend films by comparing them under N_2_ and O_2_ atmospheres as shown in Fig. S3b in the SI, with their transient decay times tabulated in Table S2. We could not detect any kind of triplet quenching in the films; hence no delayed component was observed in the transient kinetics even under N_2_ atmosphere. Coupled with the almost similar PL characteristics (except for a 6% enhancement in PLQYs for 1:3 blend films), this supported the energy transfer hypothesis. The device performances are compared and summarized in Table 1.

**Table 1:** Device performances of the blue OLEDs

Device	EML	Luminance at 10 V/(cd·m^−2^)	Current density at 10 V/(mA·cm^−2^)	EL peak/nm	Turn-on voltage/V	Maximum luminance/(cd·m^−2^)	Maximum current efficiency/(cd·A^−1^)	Maximum power efficiency/(lm·W^−1^)
B_1_	NPB/TAZ(60 nm/40 nm)	1102	303	440	3.5	1419	0.4	0.16
B_2_	NPB:TAZ(1:1, 15 nm)	1703	246	440	3.6	1941	0.75	0.36
B_3_	NPB:TAZ(1:3, 15 nm)	642	93	440	3.8	984	0.73	0.35

From the spectroscopic studies of the thin-films, we could say that we have evidence for both exciplex formation and energy transfer from TAZ to NPB. The redshifted and slightly broader spectra of the blend film, compared to the spectra for the neat films, could be evidence for exciplex formation. At the same time, favorable spectral overlap between emission of TAZ and absorption of NPB and the improved PLQY of the blend film with lower concentration of NPB can be cited as supporting evidence for energy transfer from TAZ to NPB. However, the lack of delayed emission for the blend film compared to the neat films could be evidence of no exciplex formation. Hence, it appears that there is a complex mix of different mechanisms.

From the performance of blue devices, it is clear that blend devices exhibit better performance compared to the bilayer device, which may be supporting the argument in favor of exciplex formation. Bulk exciplexes are reported to be more stable than interface exciplex as more intermolecular exciplexes can be formed in the bulk, compared to the interface [17]. Also, in this study, the device with 1:1 ratio between NPB and TAZ worked better than the device with 1:3 ratio. In general, exciplexes work best with 1:1 ratio [11]. This also supports the formation of an exciplex between NPB and TAZ. However, we do not completely rule out energy transfer, though NPB is a weak emitter. In fact, both mechanisms may co-exist also.

#### Yellow and white OLEDs using phosphorescent dopant in the NPB:TAZ exciplex host

**Fig. 3:**
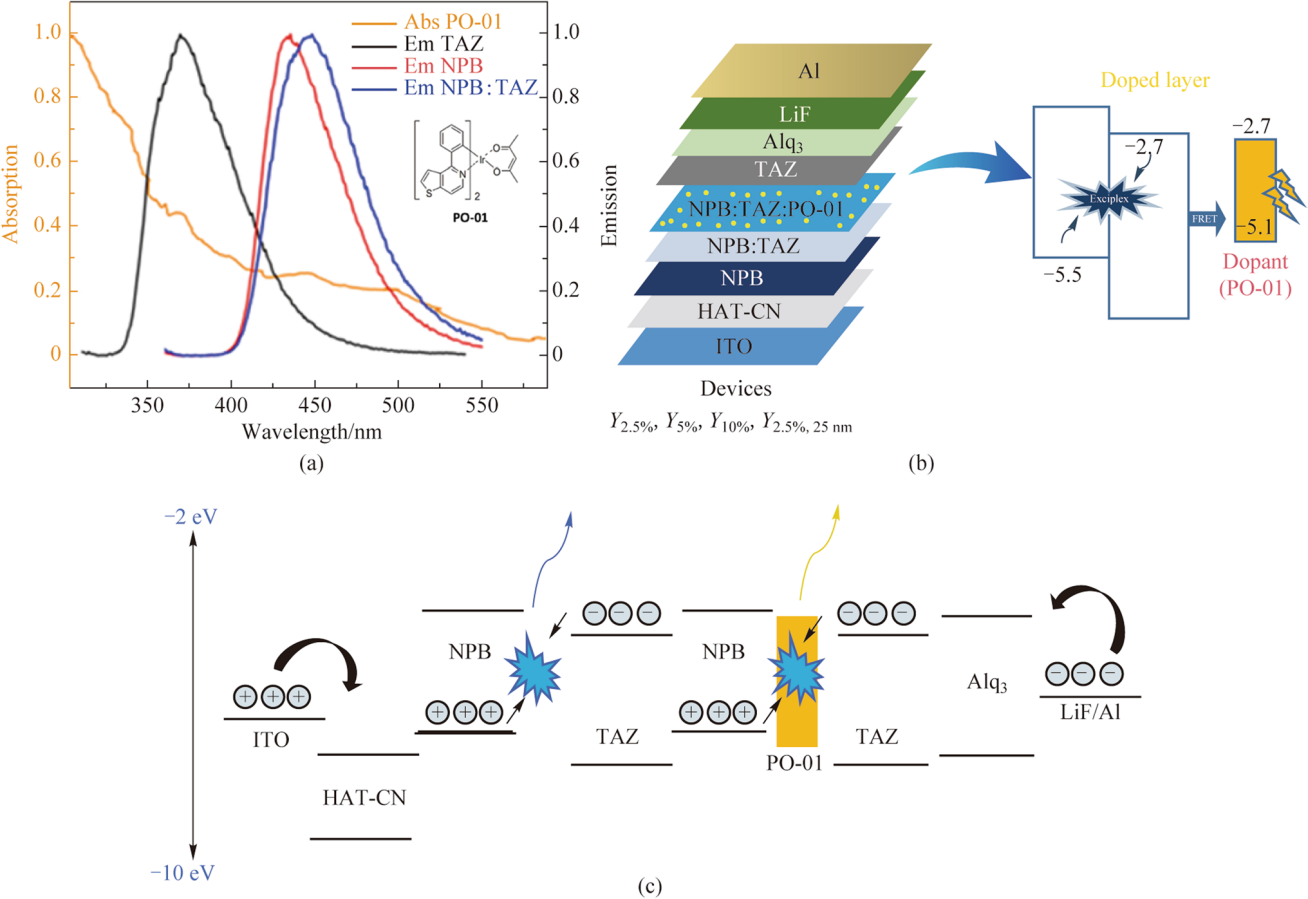
**a** Spectral overlap between emission of NPB and TAZ with absorption of dopant (PO-01), with the chemical structure of PO-01 shown inset). **b** Device architecture (NPB:TAZ doped with PO-01 at 2.5%, 5% and 10%, respectively). **c** Energy level diagram showing the mechanism responsible for the yellow emission

**Fig. 4:**
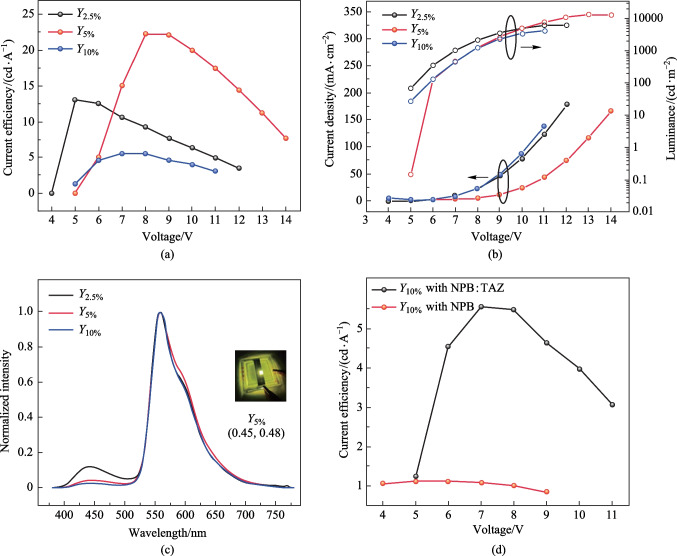
**a** Current efficiency vs voltage plots for the yellow devices. **b** *J–V–L* plots. **c** EL spectra of the yellow devices. **d** Current efficiency vs voltage plots for devices with NPB and NPB:TAZ as host with 10% of PO-01

The core idea of this work was to develop novel device design strategies to improve the quality of white light in OLEDs using cost-effective solutions. A mixed host system can effectively transfer its energy to the dopants via Förster or Dexter energy transfer mechanisms. Therefore, a dopant was selected based on the spectral overlap of the emission of blend films of NPB:TAZ and the absorption of the dopant. Hence, a yellow phosphorescent dopant, PO-01, was studied as the emitter with our NPB:TAZ host as the yellow OLEDs. Figure 3a shows the spectral overlap of PO-01 and NPB:TAZ films and Fig. 3b depicts the yellow OLED device architecture, consisting of a co-evaporation of PO-01, NPB and TAZ as the EML, sandwiched by NPB and TAZ layers. The emissive layer consisted of a mixed layer NPB:TAZ (1:1, 10 nm) followed by the emissive layer of NPB:TAZ:PO-01 (1:1, *x*%, 5 nm), where *x* = 2.5%, 5% and 10% for devices, *Y*_2.5%_, *Y*_5%_ and *Y*_10%_, respectively. The energy level diagram is shown in Fig. 3c. It was found that device *Y*_5%_ showed the best performance with maximum brightness of 13,070 cd/m^2^ and an EQE of (6.9 ± 0.27)% @ 1000 cd/m^2^ (Fig. 4). The current efficiency vs voltage and *J–V–L* plots for the yellow devices are shown in Fig. 4a, b. Figure 4c compares the EL spectra of the devices, dominated by a yellow emission at around 560 nm, in addition to a blue emission at around 444 nm. The yellow emission is likely due to an energy transfer from the exciplex to PO-01. When the dopant concentration was decreased to 2.5%, the relative contribution of the exciplex slightly enhanced to give a warm white emission with CIE coordinates (0.43, 0.46) with a blue to yellow emission ratio of 12%. The summary of device performance of yellow OLEDs is tabulated in Tables 2 and 3 and Table S4 in the SI (with error bars). This device is more promising regarding white emission, but the blue emission is weak. To further enhance the blue contribution, we increased the thickness of the NPB:TAZ layer to 25 nm. Hence, a new device *Y*_2.5%_ with 25 nm blue EML as NPB:TAZ (1:1, 25 nm)/NPB:TAZ:PO-01(1:1,2.5%, 5 nm) was fabricated. It has been reported that the excitons in charge transfer (CT) states in D-A blends can diffuse much more than Frenkel excitons [18]. However, the ratio of blue to yellow emission remained at 7% with the total brightness falling compared to the device *Y*_2.5%_. To further study the thickness dependence of blue layer, we again increased the blue EML from 25 to 30 nm. The white quality improved but the device efficiency drastically reduced. The comparison of the current efficiency values with voltages and comparison of EL plots are given in Fig. S4a and b in the SI.

**Table 2:** Summary of device performance of yellow OLEDs

Device	EML	Luminance at 11 V/(cd·m^−2^)	cd/A (@ 1000 cd· m^−2^)	Turn-on voltage/V	Maximum luminance/(cd·m^−2^)	Maximum current efficiency/(cd·A^−1^)	Maximum power efficiency/(lm·W^−1^)	Maximum EQE/%
*Y*_2.5%_	NPB:TAZ(10 nm)/NPB:TAZ:PO-01(1:1,2.5%, 5 nm)	6090	9.8	4.1	6164	13	8	4
*Y*_5%_	NPB:TAZ(10 nm)/NPB:TAZ:PO-01(1:1,5%, 5 nm)	7750	20	4.3	13,070	22	8.7	7
*Y*_10%_	NPB:TAZ(10 nm)/NPB:TAZ:PO-01(1:1,10%, 5 nm)	4240	5.47	4.8	4244	5.5	2.5	1.7
*Y*_2.5%, 25 nm_	NPB:TAZ(25 nm)/NPB:TAZ:PO-01(1:1,2.5%, 5 nm)	5024	13.9	5.9	7913	14	5.5	4.5

**Table 3:** Summary of emission parameters of yellow OLEDs

Device	EML	EL peak wavelength/nm		Ratio of blue to yellow/%
		Yellow	Blue	
*Y*_2.5%_	NPB:TAZ(10 nm)/NPB:TAZ:PO-01 (1:1,2.5%, 5 nm)	560	440	12
*Y*_5%_	NPB:TAZ(10 nm)/NPB:TAZ:PO-01 (1:1,5%, 5 nm)	560	440	4
*Y*_10%_	NPB:TAZ (10 nm)/NPB:TAZ: PO-01(1:1,10%, 5 nm)	560	440	3
*Y*_2.5%, 25 nm_	NPB:TAZ(25 nm)/NPB:TAZ:PO-01 (1:1,2.5%, 5 nm)	560	444	7

The energy level diagram in Fig. 3c shows the emission mechanisms in the yellow and blue emitting units in detail. The yellow emitting unit consists of the yellow dopant molecule in the NPB:TAZ matrix. The spectral overlap shows the chances for energy transfer from NPB:TAZ, NPB or TAZ to PO-01, yielding the yellow emission. In contrast, for charge injection, the holes and electrons should reach both NPB and TAZ for the yellow emission. However, given that there is a high energy barrier for transfer of holes from NPB to TAZ (0.8 eV) in the blend; the migration of holes toward the HOMO of NPB in the NPB:TAZ:PO-01 layer could have been hindered due to the presence of TAZ in the NPB:TAZ layer. Hence, NPB and TAZ in the NPB:TAZ:PO-O1 layer cannot be individually electrically excited easily.

To rule out the possibility of energy transfer from NPB to P0-01, we fabricated an equivalent device without TAZ where NPB was used as a host. The current efficiency vs voltage plots for devices with NPB and NPB:TAZ as host with 10% of PO-01 is shown in Fig. 4d. The device only showed green emission of Alq_3_ instead of yellow emission_._ This indicates that energy transfer from NPB to the dopant is unlikely. This also showed the direct excitation of PO-01 is unlikely in the current device structure, leaving next possibility of energy transfer from exciplex to PO-01. This indeed happened while the proposed exciplex in the NPB:TAZ layer was responsible for the blue emission.

As mentioned earlier, increasing the thickness of the NPB:TAZ layer to 25 and 30 nm to further enhance the blue contribution did not yield the expected results. The slow migration of holes from the NPB alone layer toward NPB:TAZ:PO-01 layer could have been an impediment. The low *J* value of the device shows that the increased thickness of the layer resulted only in increasing the device resistance instead of enhancing the blue emission.

The weak intensity of blue emission is primarily due to the difference in hole and electron mobilities of the component molecules. Due to the high hole mobility of NPB, more holes get accumulated at the HOMO of NPB in the NPB:TAZ layer. The comparatively lower electron mobility of TAZ and the longer path for electrons from cathode to reach this layer can delay electrons migration to the LUMO of TAZ in the NPB:TAZ layer. This loss of carriers can lead to decreased blue exciton formation and subsequent emission in the NPB:TAZ layer. A similar issue can be expected for exciplex formation in the NPB:TAZ:PO-01 layer as well. But here the effect of longer path for holes from anode and low mobility of electrons in TAZ can create a balance and hence a promising white emission is to be expected. The device architecture comprises of two emissive units with co-deposited film of NPB and TAZ. Hence there will be possibility of uncontrolled flow of carriers through the co-deposited layers. This can reduce the charge balance factor and the formation of excitons. The unbalanced flow of carriers to the NPB:TAZ layer might be a reason for reduction in blue emission among the white devices. To study the problem of carrier imbalance in the device, we fabricated the hole-only and electron only devices. The device architecture was as follows: ITO/HAT-CN(5 nm)/NPB(60 nm)/NPB:TAZ(50 nm)/Ag(100 nm) for the hole-only device and Al(100 nm)/LiF(1 nm)/BCP(5 nm)/NPB:TAZ(50 nm)/TAZ(40 nm)/Alq_3_(20 nm)/LiF(1 nm)/Al(100 nm) for the electron-only device. The comparison of the *J–V* characteristics of the devices are shown in Fig. S5 in the SI. The hole-only devices showed much higher current density compared to electron only devices; this observation is evidence for the charge imbalance in the NPB:TAZ mixed layer.

#### White OLEDs using charge generation layer and tetracene spacer

To further improve the quality of white emission, it is critical to balance the blue and yellow emission This can be done by adjusting the flow of holes and electrons toward the respective layers by incorporating a charge generation layer (CGL) between the two emitting units. A suitable CGL would provide adequate flow of electrons and holes toward the respective units. We have therefore selected a typical fullerene (C_60_)/pentacene organic heterojunction as the CGL [19]. The high electron mobility of C_60_ [20] and high hole mobility of pentacene [21] can make this p–n junction an efficient CGL [22]. The balanced flow of electrons from C_60_ toward the NPB:TAZ layer can be expected to enhance the blue emission. Also, the availability of holes in the NPB:TAZ:PO-01 layer is ensured by pentacene. The EML of devices with CGL has the structure, NPB:TAZ (1:1, 10 nm)/C_60_ (15 or 10 nm)/pentacene (10 or 5 nm)/NPB:TAZ:PO-01 (5 nm, 2.5% PO-01). The device architecture and the energy level diagram are given in Figs. S6a and b in the SI.

**Fig. 5:**
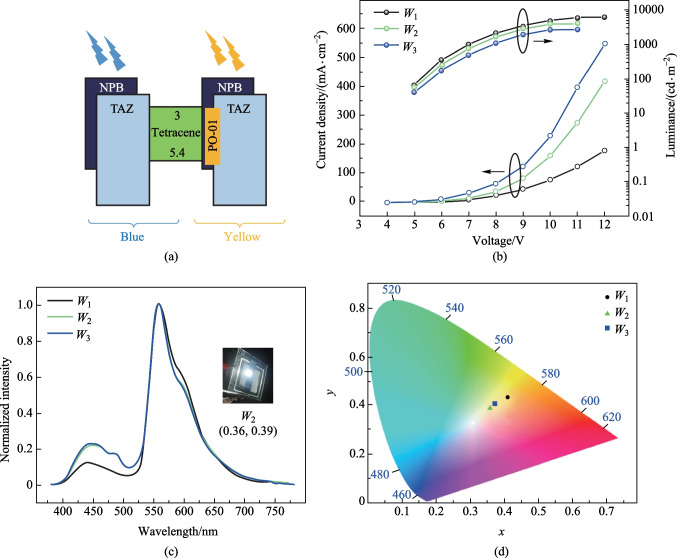
**a** Energy level diagram of white OLEDs with spacer. **b** *J–V–L* characteristics. **c** EL characteristics (photograph of the white OLED (*W*_2_) with its CIE coordinates is shown as inset). **d** CIE diagram

The thickness of C_60_ was kept slightly higher than that of pentacene to compensate the high hole mobility of pentacene [21] compared to the electron mobility of C_60_. However, the device performance was drastically diminished upon the incorporation of the CGL. The device *J–V–L* and EL characteristics of the devices are shown in Fig. S6c and d in the SI. Hence, unlike in a normal tandem WOLED, the CGL here functioned more like a barrier. The increase in total device thickness might also have contributed to the poor performance. However, the percentage of blue emission slightly improved in these devices compared to the same percentages without a CGL. This implies the need of a separation layer other than CGL for balanced flow of carriers between the NPB:TAZ:PO-01 and NPB:TAZ units of exciplex, which can be called as a spacer layer. In this context, it was further proposed that an ambipolar spacer material would be a better choice than a p–n junction. The energy levels of the spacer material should be conducive to not completely blocking electrons and holes. We, therefore, selected tetracene as the spacer layer as it met the above requirements. A thin layer of tetracene was used as a spacer layer between the NPB:TAZ (blue emitter) and NPB:TAZ:PO-01 (yellow emitter) layers. The energy level diagram of the emissive units for the modified device architecture for WOLED is shown in Fig. 5a. The devices with tetracene blocking layer indeed resulted in a higher intensity of blue emission compared to the CGL devices. The weak peak at about 484 nm could be the monomer emission of tetracene [23]. The electroluminescence of tetracene film was around 530 nm [24], which was absent in the EL spectra. Hence, the possibility of emission from the spacer layer can be ruled out. We took the device *Y*_2.5%_ as the reference device for white emission, and this device is now designated as *W*_1_. The EML of device *W*_2_ had the structure NPB:TAZ (1:1, 10 nm)/tetracene (5 nm)/NPB:TAZ:PO-01 (5 nm, 2.5% PO-01). Increasing the thickness of tetracene layer to 10 nm (Device *W*_3_) did not improve the performance. However, tetracene devices showed a better performance compared to performances with CGL. The poor performance of the 10 nm spacer layer compared to that of the 5 nm shows the impact of resistance in the devices. We have earlier seen that the CGL devices also fared poorly after the total device thickness increased after the insertion of the CGL. The *J–V–L* and EL characteristics are shown in Fig. 5b, c and the device performances for WOLEDs with spacer layer are summarized in Table 4. We achieved a white emission with CIE coordinates of (0.36, 0.39), when 5 nm of tetracene was employed. The CIE diagram for WOLEDs is shown in Fig. 5d. The ratios of intensities of blue and yellow emissions were compared. The intensity of blue emission was increased from 12% to 23%, when the spacer layer was employed. This could be attributed to the balanced flow of carriers to NPB:TAZ layer due to the ambipolar nature of tetracene layer. The increase in current density after the addition of tetracene layer is evidence for the role of tetracene in the charge transport mechanism in the device. Unlike in CGL, a single layer can provide improved white light as well as device performance. The efficiency comparison of the WOLEDs with CGL and spacer layer is shown in Table S3 in the SI. Hence, device architecture with a spacer layer can be considered as an alternative to complicated tandem structures. A balanced white OLED combining yellow emission from dopant and blue emission from exciplex was achieved with an ambipolar thin spacer layer.

**Table 4:** Summary of device performances of the WOLEDs

Device	EML	Luminance at 11 V/(cd·m^−2^)	cd/A (@ 1000 cd·m^−2^)	Turn-on voltage/V	Maximum luminance/(cd·m^−2^)	Maximum current efficiency/(cd·A^−1^)	Maximum power efficiency/(lm·W^−1^)	Maximum EQE/%
*W*_1_	NPB:TAZ(10 nm)/NPB:TAZ:PO-01 (1:1,2.5%, 5 nm)	6090	9.8	4.1	6164	13	8	4
*W*_2_	NPB:TAZ(10 nm)/Tetracene(5 nm)NPB:TAZ: PO-01(1:1,2.5%, 5 nm)	4040	4.6	4	4043	6	3.8	2
*W*_3_	NPB:TAZ(10 nm)/Tetracene(10 nm)NPB:TAZ: PO-01(1:1,2.5%, 5 nm)	4240	1.7	4	2675	2	1	0.6

## Conclusion

A novel blue emitting exciplex system, utilizing commonly used charge transporting materials, NPB and TAZ, is presented. This intermolecular exciplex used as a blue emitter as well as a host for a yellow dopant to afford white light. Yellow OLEDs were fabricated by using a mixed host with a phosphorescent dopant (PO-01). An EQE of (6.9 ± 0.27)% @ 1000 cd/m^2^ was obtained for the yellow OLED, with 5% of PO-01 doped into the NPB:TAZ matrix. White emission with CIE coordinates (0.36, 0.39) and color temperature of 4643 K was achieved by using a novel device design, employing tetracene as a spacer, to balance the carrier transport. The strategy presented here may be utilized for creating tailor-made molecules to realize stable and efficient blue emission, with device designs other than complicated tandem structures for obtaining white emission.

### Supplementary Information

Below is the link to the electronic supplementary material.

Supplementary file1 (PDF 865 KB)

## Data Availability

The data that support the findings of this study are available from the corresponding author, upon reasonable request.
