# Supplementary material for: Blue emitting exciplex for yellow and white organic light-emitting diodes
Source: Front Optoelectron. 2023 Dec 14;16(1):46. doi: 10.1007/s12200-023-00101-3 (PMC10721783; doi:10.1007/s12200-023-00101-3)
Supplement: Supplementary file 1 — Supplementary file1 (PDF 865 KB) [file 12200_2023_101_MOESM1_ESM.pdf]

Additional file 1

Blue emitting exciplex for yellow and white organic light-emitting diodes

Kavya Rajeev[1,2], C. K. Vipin[1,2], Anjali K. Sajeev[1,2], Atul Shukla[3,4], Sarah K. M. McGregor[3,5], Shih-Chun Lo[3,5], Ebinazar B. Namdas[3,4], K. N. Narayanan Unni[1,2]*

1 Centre for Sustainable Energy Technologies, CSIR-National Institute for Interdisciplinary Science and Technology, Thiruvananthapuram, 695 019, India

2 Academy of Scientific and Innovative Research (AcSIR), Ghaziabad 201002, India

Atul Shukla, Ebinazar B. Namdas

3 Centre for Organic Photonics & Electronics, The University of Queensland, Brisbane, QLD 4072, Australia

4 School of Mathematics and Physics, The University of Queensland, Brisbane, QLD 4072, Australia

5 School of Chemistry and Molecular Biosciences, The University of Queensland, Brisbane, QLD 4072, Australia

Corresponding author: K.N. Narayanan Unni, E-mail: unni@niist.res.in

© The author(s) 2023

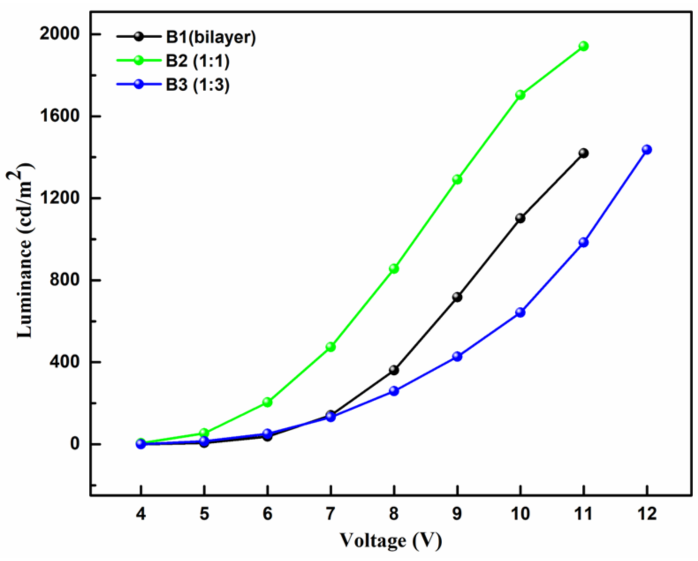

Fig S1. Luminance Vs current density plots of the blue devices

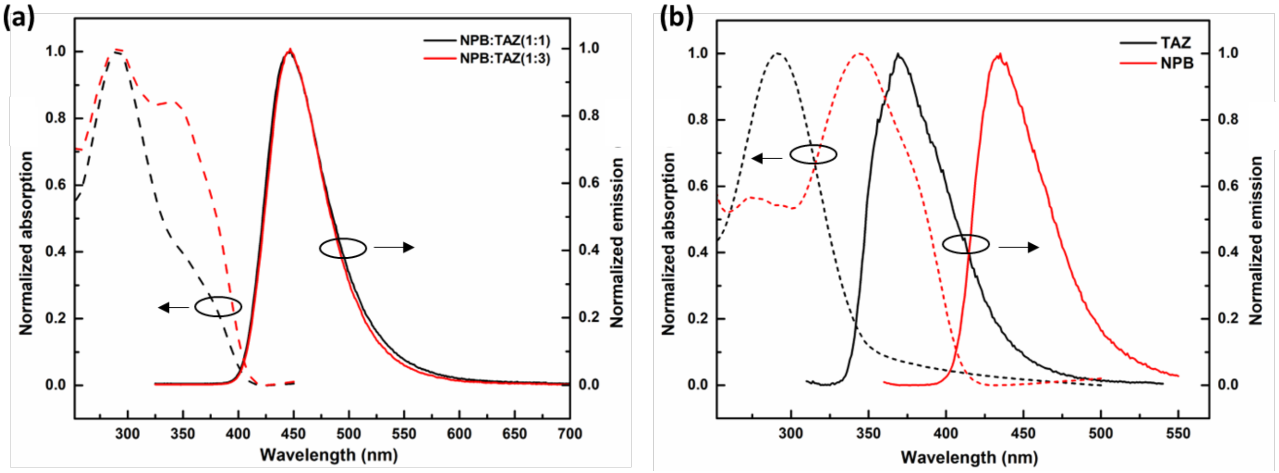

Fig S2. The UV-Visible absorption spectra as well as emission spectra of the films of (a) NPB:TAZ (1:1, 1:3) (b) TAZ and NPB

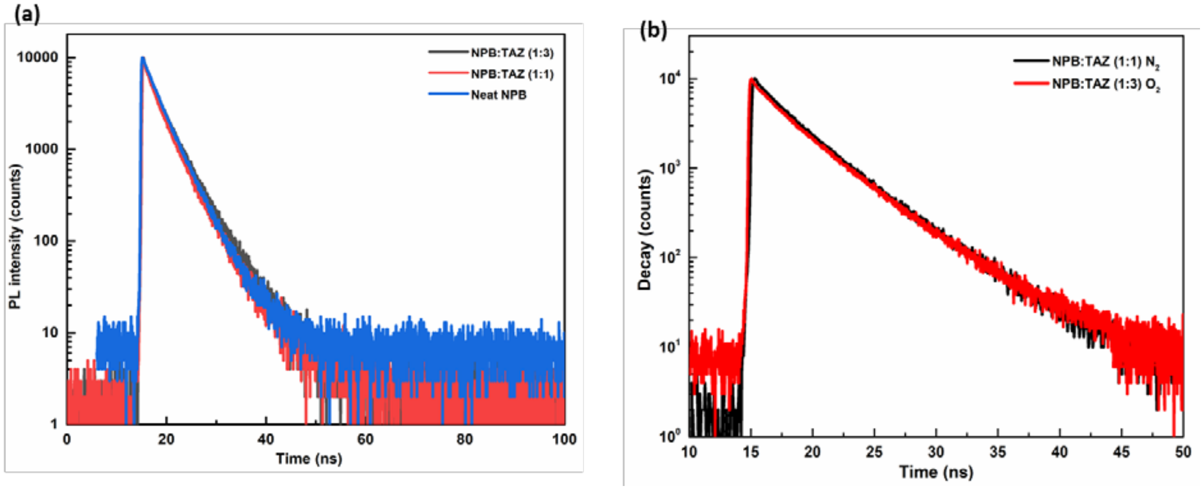

Fig S3. The transient emission properties of the **(a)** neat NPB and mixed films of NPB:TAZ (1:1, 1:3), (b) mixed films in N_2 and O_2

Table S1. fluorescence decay parameters of NPB, TAZ and NPB:TAZ films (**τ**: Fluorescence lifetime decay (ns), **PLQY** : photoluminescence quantum yield, k_r : radiative decay rate(s^{-1}), k_{nr} : non-radiative decay rate(s^{-1}) , **FWHM** : Full width at half maximum(nm)) Comparison of transient studies of NPB:TAZ films.

	τ_1 (ns)	τ_2 (ns)	τ_{avg} (ns)	PLQY (%)	k_r ($\times 10^8$) (s^{-1})	k_{nr} ($\times 10^8$) (s^{-1})	FWHM (nm)
**NPB neat film**	2.2	4.1	3.5	31	0.88	1.97	52
**TAZ neat film**	-	-	-	68	-	-	54

**NPB:TAZ (1:3)**	1.96	4.5	3.8	44	1.15	1.46	63
**NPB:TAZ (1:1)**	1.81	4.4	3.5	38	1.09	1.77	60

Table S2. Comparison of fluorescence decay parameters of NPB:TAZ films under N_2 and O_2

	**Under N_2**			**Under O_2**		
	**τ_1 (ns)**	**τ_2 (ns)**	**τ_{avg} (ns)**	**τ_1 (ns)**	**τ_2 (ns)**	**τ_{avg} (ns)**
**NPB:TAZ (1:3)**	1.96	4.5	3.8	1.9	1.44	3.8
**NPB:TAZ (1:1)**	1.81	4.4	3.5	1.77	4.25	3.4

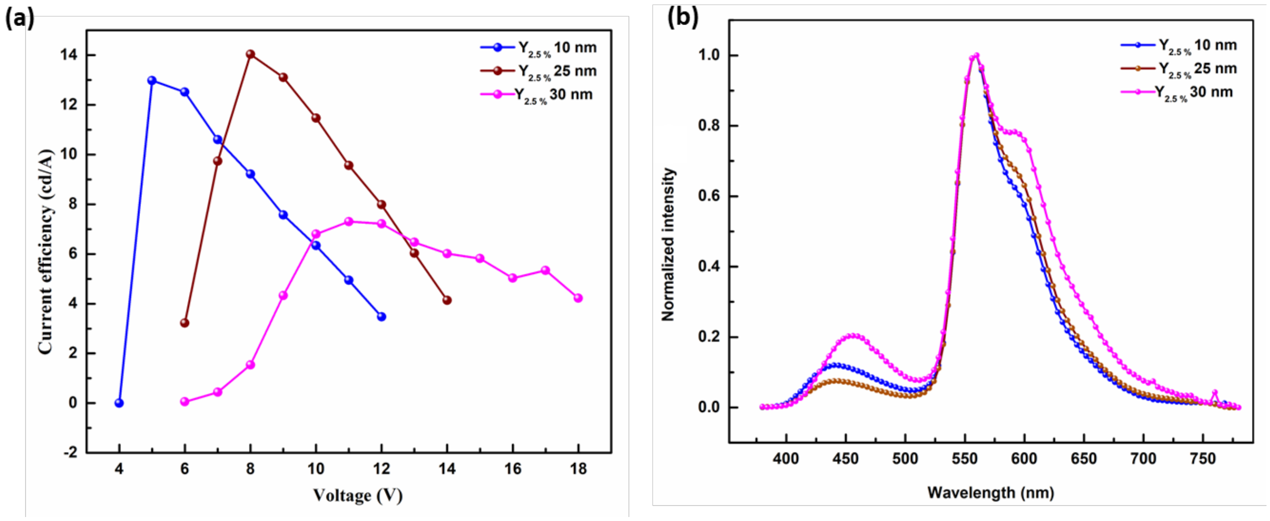

Fig S4. (a) Current efficiency Vs Voltage plot and (b) EL characteristics of the devices $Y_{2.5\%}$,10nm, $Y_{2.5\%}$, 20 nm and $Y_{2.5\%}$, 30 nm

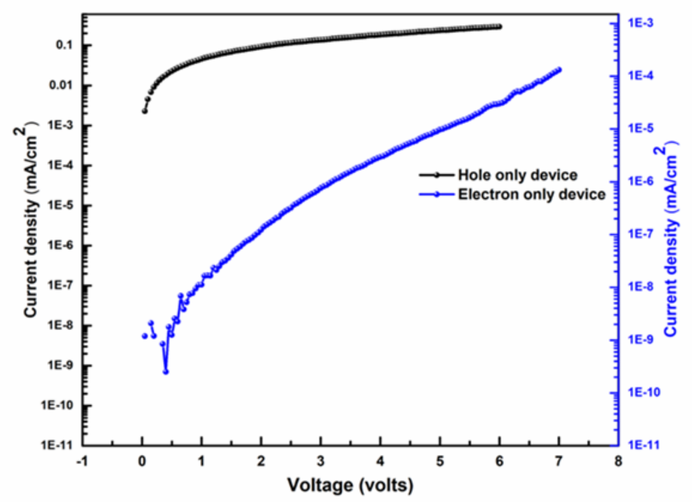

Fig S5. Current density vs. Voltage plots for the electron only and hole only devices

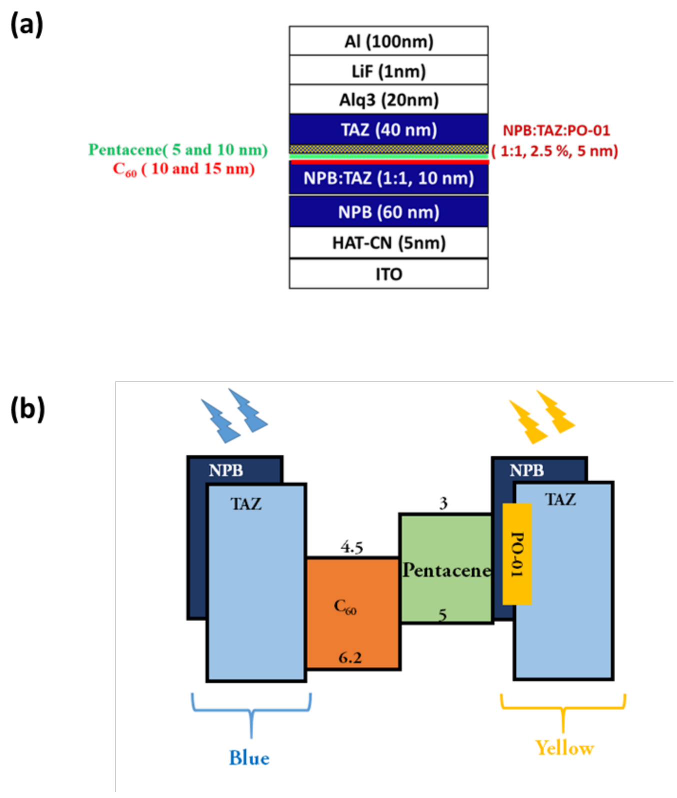

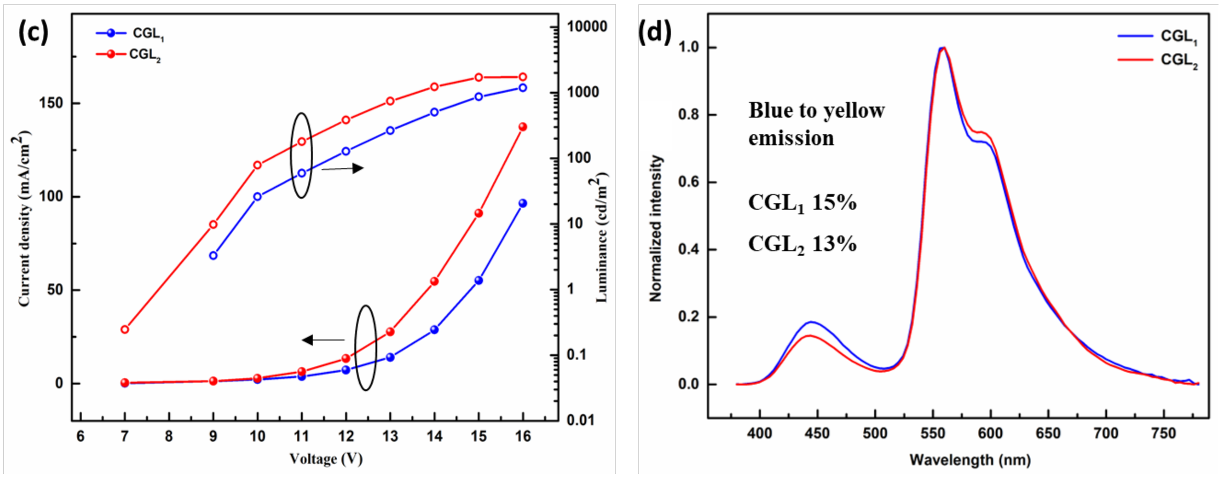

Fig S6. (a) Device structure and (b) Energy level diagram of devices with CGL (CGL$_1$: C_{60} (10nm)/Pentacene(5nm), CGL$_2$: C_{60} (15nm)/Pentacene(10nm) (c) J-V-L and (d) EL characteristics for devices with CGL

Table S3. Efficiency comparison of WOLEDs with CGL and spacer layer (**CE** : Current Efficiency, **PE** : Power Efficiency)

Devices	EML	CE (cd/A) at 1000cd/m^2	CE (cd/A) at 10 mA/cm^2	PE (lm/W) at 10 V
**CGL$_1$**	C_{60}(10 nm)/Pentacene (5 nm)	1.5	1.7	0.4
**CGL$_2$**	C_{60}(15 nm)/Pentacene (10 nm)	1.2	2.8	0.9
**W$_2$**	Tetracene (5nm)	4.7	5.2	0.7
**W$_3$**	Tetracene (10nm)	1.6	1.6	0.4

Table S4. The summary of device performance of yellow OLEDs

Device	EML	Luminance @ 11 V (cd/m^2)	Current density @ 11 V (mA/cm^2)	Cd/A @ 11 V	EQE @ 11 V
$\mathbf{Y_{2.5\%}}$	NPB:TAZ(10nm)/NPB:TAZ: PO-01(1:1,2.5 %, 5 nm)	6088 ± 116	123 ± 4.53	5 ± 0.56	1.6 ±0.13
$\mathbf{Y_{5\%}}$	NPB:TAZ(10nm)/NPB:TAZ: PO-01(1:1,5 %, 5 nm)	7747 ± 129	44 ± 3.16	17.4 ± 0.15	5 ± 0.35
$\mathbf{Y_{10\%}}$	NPB:TAZ (10nm)/NPB:TAZ: PO-01(1:1,10 %, 5 nm)	4240 ± 330	138 ± 7.49	3.06 ± 0.03	0.8 ± 0.007
$\mathbf{Y_{2.5\%,}}$ **25 nm**	NPB:TAZ(25nm)/NPB:TAZ: PO-01(1:1,2.5 %, 5 nm)	5024 ± 387	52±1.17	9.6 ± 1.2	3 ±0.18
